# Burden of Maternal and Neonatal Disorser in Nepal from 1990 to 2019; Analysis of Data from Global Burden of Disease Study: An Observational Study

**DOI:** 10.31729/jnma.8916

**Published:** 2025-03-31

**Authors:** Pramila Rai, Ilana N. Ackerman, Denise A. O'Connor, Ganesh Dangal, Rachelle Buchbinder

**Affiliations:** 1 School of Public Health and Preventive Medicine, Monash University, Melbourne, Victoria, Australia; 2 Kathmandu Model Hospital, Exhbition Road, Kathmandu, Nepal

**Keywords:** *global burden of disease*, *infant, newborn, diseases*, *maternal health*, *Nepal*

## Abstract

**Introduction:**

Nepal continues to face significant challenges with high maternal and neonatal mortality. To improve health and achieve the Sustainable Development Goals of reducing maternal and newborn mortality by 2030, Nepal needs to focus on addressing high-burden maternal and neonatal disorders. The objective of the study was to examine the current burden of maternal and neonatal disorders in Nepal and to assess any changes over time.

**Methods:**

We examined the annual Global Burden of Disease Study data on prevalence, deaths, Years Lived with Disability, and Disability-Adjusted Life Years for maternal and neonatal disorders in Nepal for the 1990-2019 period. Estimated annual percentage changes were also calculated to assess the trends of the age-standardised rates of these burden metrics.

**Results:**

The analysis found that the prevalence of overall maternal disorder in Nepal decreased by 37% from 128,176 in 1990 to 80,724 in 2019 with Age-Standarised Ratio of 432.07 per 100,000 in 2019 and Estimated Annual Percentange Change of -4.34 (CI 95%: -4.49 to -4.18). Similarly, the overall prevalence of neonatal disorder increased by 57% from 303,146 in 1990 to 475,544 in 2019 with Age-Standarised Ratio of 1521.14 per 100,000 in 2019 and Estimated Annual Percentage Change of 0.98 (95% CI: 0.67-1.29).

**Conclusions:**

Our findings emphasise the need to address maternal haemorrhage, indirect maternal deaths, maternal abortion and neonatal disorders in Nepal in future national health programs.

## INTRODUCTION

Nepal initiated the national Safe Motherhood program in 1993 to improve maternal healthcare services. The Millennium Development Goals of reducing the maternal mortality ratio (MMR) and Neonatal mortality rate (NMR) further accelerated these efforts.^1,2^ Consequently, MMR dropped from 539 per 100,000 live births in 1998 to 151 in 2022,^3^ and NMR fell from 50 per 1,000 live births in 1996 to 21 in 2022.^4^ However, Nepal still has the third-highest maternal and neonatal mortality in South Asia after Pakistan and Afghanistan.^3,5^

Achieving the Sustainable Development Goals of reducing the MMR to below 70 per 100,000 live births and NMR to 12 or fewer per 1,000 live births by 2030,^3,6^ requires significant improvements in maternal and neonatal health. Achieving these targets requires the identification of priority maternal and neonatal disorders and increased investment in priority areas. However, a comprehensive understanding of the current burden of these disorders and how they have evolved remains limited in Nepal.

This study aimed to assess the current burden of these disorders and examine their trend over time.

## METHODS

This is a descriptive analysis of available Global Burden of Disease study 2019 data for the burden of maternal and neonatal disorders in Nepal.^7^

### Study Population (Global Burden of Disease Study 2019 framework)

The GBD Study provides a comprehensive analysis of 286 causes of death, 369 diseases and injuries, and 87 risk factors in 204 countries and territories, using a standardised approach.^7,8^ These data are publicly available and a detailed description of the metrics, data sources and statistical modelling used in the GBD 2019 Study has been reported previously.^7^ For this study, we extracted data specific to Nepal, ensuring that the findings are representative of the Nepali Population.

### Disease definitions

**Figure 1:**
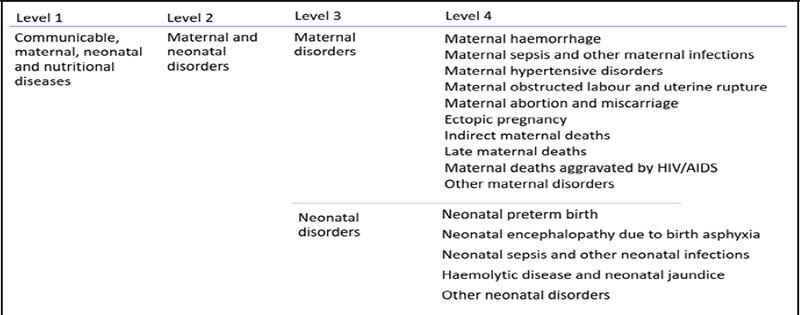
Levelled hierarchy of maternal and neonatal disorders in the Global Burden of Disease Study

Diseases are organised into a levelled cause hierarchy in the GBD Study. The levelled cause hierarchy for maternal and neonatal disorders is shown in Figure 1. We focused on the burden of Level-2 causes (maternal and neonatal disorders) and the more specific Level-3 and Level-4 causes/subcategories.^7^ The GBD maternal disorders include pregnancy complications, childbirth and the immediate postpartum period. Neonatal disorders include complications from poor fetal development, birth injuries and other disorders unique to the perinatal period. Both disorders were classified according to the 10^th^ revision of the International Classification of Disease and Injuries discharge diagnosis codes.^7^

### Sources of data for Nepal

Data for estimating the burden of maternal and neonatal disorders in Nepal in the GBD study were obtained from national and subnational sources including (but not limited to) the Nepal Demographic and Health Survey, verbal autopsy data, Nepal Department of Health Services annual report, Nepal Maternal Mortality and Morbidity study and the Nepal Population and Housing Survey (Supplementary Box S1). In populations with incomplete vital registration, verbal autopsy is employed to ascertain the causes of deaths; a trained interviewer asks questions about the demographics, symptoms and signs of a recently deceased person.^9^

### Burden of disease metrics

For each condition of interest, the GBD Study reports data on prevalence, years lived with disability (YLDs), deaths and disability-adjusted life years (DALYs).^8^ Prevalence refers to the proportion of the population with the specified condition at a specified time point. The YLDs refer to years of life lived with disorder-related disability over time, calculated as the product of the disability weight and prevalence of the disease. The disability weight is an assigned value determined by expert GBD teams between 0 and 1 representing health loss severity related to a particular disease condition. DALYs represent a composite metric combining years of life lost (YLLs) and YLDs.

### Data collection, management and statistical analysis

For this analysis, we extracted annual data for 1990-2019 from the Global Health Data Exchange query tool. Data on the absolute number, percentage change, and age-standardised rate (ASR) of prevalence, deaths, YLDs, and DALYs for maternal and neonatal disorders and level-4 subcategories were extracted including their 95% uncertainty intervals (UIs). In the GBD Study, the 95% UIs are calculated using 1000 draws from the posterior distribution at each step in the estimation process following the GBD methodology.

The lower and upper UIs are determined as the 25^th^ and 975^th^ ranked values, respectively from the 1000 ordered draws.^9^ We calculated the estimated annual percentage change (EAPC) of ASRs to illustrate average annual trends in these rates ASRs over the time period of interest. EAPCs were calculated by fitting a least squares regression line to the natural logarithmic rates, using the calendar year as a regressor variable using R software.^10^ We used the regression model: Y= a+pX+e, where Y refers to natural log of ASR, X represents the calendar year, and the e error term to estimate EAPC. The EAPC was given by 100x[exp(P)-1], along with the 95% confidence interval (CI) with the linear regression model. A negative EAPC indicated a decreasing trend over time, while a positive EAPC indicated an increasing trend. Expedited review was obtained from Nepal Health Research Council (Ref. No. 1600).

## RESULTS

**Figure 2:**
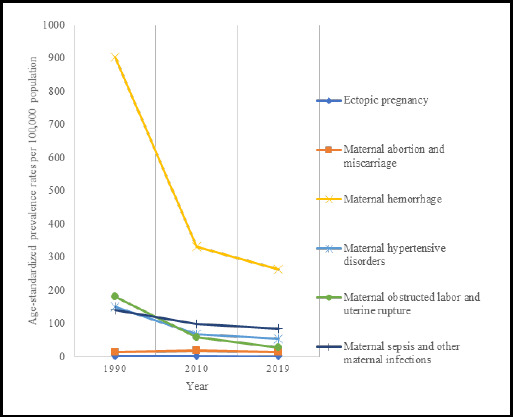
The trend of age standardised prevalence rate per 100,000 population

In Nepal, the prevalence of overall maternal disorder decerased by 37% from 128,176 in 1990 to 80,724 cases in 2019. The ASR for prevalence of maternal haemorrhage was 262.20/100,000 and 85.29/100,000 for maternal sepsis in 2019 (Table 1, Figure 2). The EAPC for overall maternal disorders was -4.34 (95% CI: -4.49 to -4.18), (Table 1).

**Figure 3:**
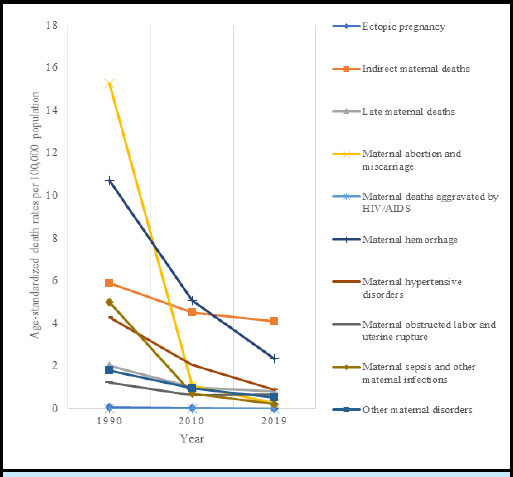
The trend of age standardised death rate per 100,000 population

The number of deaths due to overall maternal disorders decreased by 59% from 4,145 in 1990 to 1,714 in 2019. The ASR of death for the indirect deaths resulting from previous existing diseases or diseases that developed during pregnancy and aggravated by physiologic effects of pregnancy such as cardiovascular diseases was 4.11/100,000 and 2.36/100,000 for maternal haemorrhage in 2019 (Table 1, Figure 3, Supplementary Figure S1). The EAPC for deaths due to overall maternal disorders was -5.04 (95% CI: -5.38 to -4.70), (Table 1).

The YLDs due to overall maternal disorders decreased by 51% from 10,442 in 1990 to 5,155 in 2019. The ASR of YLD for maternal obstructed labour was 9.35/100,000 and 8.07/100,000 for maternal hemorrhage in 2019. The EAPC of YLDs for overall maternal disorder was -4.97 (95% CI: -5.03 to -4.92), (Table 1).

The DALYs due to overall maternal disorders decreased by 59% from 2,52,170 in 1990 to 1,02,859 in 2019. Indirect maternal deaths had the DALY ASR of 234.48/100,000 and 133.85/100,000 for maternal haemorrhage in 2019 (Table 1). The EAPC for DALYs of overall maternal disorder was -5.11 (95% CI: -5.42 to -4.80), (Table 1).

The prevalence of neonatal disorder increased by 57% from 303,146 in 1990 to 475,544 cases in 2019. The ASR for prevalence of preterm birth was 1263.14/100,000 and 286.53/100,000 for neonatal sepsis in 2019 (Table 2, Supplementary Figure S2). The EAPC for overall neonatal disorders was 0.98 (95% CI: 0.67 to 1.29), (Table 2).

Deaths due to neonatal disorders decreased by 67% from 25,212 in 1990 to 8,397 in 2019. The ASR of death for other neonatal disorders ( not categorize specifically) was 13.61/100,000 and 5.23/100,000 for neonatal encephalopathy in 2019 (Table 2, Supplementary Figure S3 and S4). The EAPC for death in overall neonatal disorders was -2.61 (95% CI: -2.87 to -2.35), (Table 2).

The YLDs due to overall neonatal disorders increased by 110% from 55,716 in 1990 to 117,301 in 2019. The ASR of YLDs for preterm birth was 221.64/100,000 and 13.53/100,000 for hemolytic disease and other neonatal jaundice in 2019. The YLDs for overall neonatal disorders was 2.30, (95% CI: 1.95 to 2.65), (Table 2).

The DALYs due to overall neonatal disorders decreased by 62% from 2,295,550 in 1990 to 863,367 in 2019. The ASR of DALYs for other neonatal disorders was 1,228.50/100,000 and 498.35/100,000 neonatal encephalopathy in 201. The EAPC for overall neonatal disorders was -2.29 (95% CI: -2.49 to -2.08), (Table 2)

**Table 1:** All-age years lived with disability, disability-adjusted life years and ASR for maternal disorders in Nepal, 1990-2019

Disease category and burden of disease metric	Number in 1990 (95% UI)	Number in 2019 (95% UI)	% Change from 1990-2019 (95% UI)	ASR^*^ 1990 (per 100,000) No. (95% UI)	ASR^*^ 2019 (per 100,000) No. (95% UI)	EAPC^**^ (95% CI)
**Maternal disorders**						
Prevalence	128,176 (104,906, 156,822)	80,724 (65,949, 96,732)	-0.37 (-0.46, -0.27)	1,363.24 (1,123.04, 1,654.94)	432.07 (354.7, 514.83)	-4.34 (-4.49, -4.18)
Deaths	4,145 (3195, 5216)	1,714 (1203, 2299)	-0.59 (-0.73, -0.4)	46.27 (46.27, 59.07)	9.89 (6.94, 13.16)	-5.04 (-5.38, -4.7)
YLDs	10,442 (7,084, 14,377)	5,144 (3,392, 7,159)	-0.51 (-0.59, -0.42)	117.30 (79.77, 162.94)	28.2 (18.64, 39.16)	-4.97 (-5.03, -4.92)
DALYs	252,170 (252,170, 314,940)	102,859 (73,514, 136,751)	-0.59 (-0.73, -0.42)	2,747.18 (2,747.18, 3,444.58)	581.15 (414.71, 772.79)	-5.11 (-5.42, -4.80)
**Maternal haemorrhage**						
Prevalence	86,569 (66,307, 112,850)	49,695 (39,374, 61,374)	-0.43 (-0.52, -0.31)	902.22 (693.45, 1173.64)	262.20 (208.6, 321.24)	-4.82 (-5.06, -4.58)
Deaths	926 (673, 1259)	394 (262, 541)	-0.57 (-0.74, -0.35)	10.70 (7.79, 14.57)	2.36 (1.57, 3.29)	-4.53 (-4.85, -4.2)
YLDs	3,548 (2,191, 5,296)	1,527 (918, 2,281)	-0.57 (-0.67, -0.45)	37.00 (22.74, 55.23)	8.07 (4.83, 12.15)	-5.82 (-6.11, -5.54)
DALYs	56,061 (41,459, 74,882)	22,943 (15,883, 30,891)	-0.59 (-0.74, -0.39)	628.45 (469.24, 839.47)	133.85 (92.28, 179.75)	-4.74 (-5.04, -4.44)
**Maternal sepsis and other maternal infections**						
Prevalence	13,625 (8438, 20,712)	15,339 (10,625, 21,608)	0.13 (-0.01, 0.31)	141.45 (92.68, 209.47)	85.29 (61.33, 117.22)	-1.97 (-2.04, -1.89)
Deaths	463 (333, 630)	39 (25, 55)	-0.92 (-0.95, -0.87)	5.00 (3.57, 6.78)	0.23 (0.15, 6.78)	-2.86 (-2.99, -2.74)
YLDs	520 (236, 973)	471 (211, 881)	-0.09 (-0.31, 0.17)	5.03 (2.34, 9.35)	2.42 (1.08, 4.51)	-2.86 (-2.99, -2.74)
DALYs	28,259 (20,404, 38,232)	2,704 (1847, 3717)	-0.90 (-0.94, -0.86)	296.83 (214.69, 400.59)	14.95 (10.35, 20.63)	-10.1 (-10.31, -9.9)
**Maternal hypertensive disorders**						
Prevalence	14,316 (8,866, 21,204)	10,389 (6,603, 15,397)	-0.27 (-0.39, -0.15)	151.35 (94.46, 223.14)	54.73 (35.01, 80.69)	-3.88 (-4.07, -3.69)
Deaths	392 (299, 509)	160 (112, 216)	-0.59 (-0.75, -0.4)	4.3 (3.29, 5.58)	0.91 (0.64, 1.22)	-4.82 (-5.2, -4.45)
YLDs	711 (368, 1,171)	506 (258, 877)	-0.29 (-0.45, -0.09)	7.49 (3.87, 12.27)	2.67 (1.35, 4.55)	-3.95 (-4.15, 4.55)
DALYs	23,890 (18,295, 30,666)	9,804 (6950, 13035)	-0.59 (-0.74, -0.40)	255.07 (196.08, 328.30)	54.38 (38.94, 72.38)	-4.83 (-5.18, -4.48)
**Maternal obstructed labor and uterine rupture**						
Prevalence	14,721 (10,633,19815)	4,837 (3,467, 6,405)	-0.67 (-0.73, -0.62)	180.62 (131.78, 243.27)	28.71 (20.64, 38.15)	-6 (-6.19, -5.82)
Deaths	109 (73, 154)	116 (75, 172)	0.06 (-0.37, 0.72)	1.22 (0.81, 1.72)	0.68 (0.44, 1)	-2.2 (-2.75, -1.65)
YLDs	4,689 (2,840, 7,261)	1,578 (899, 2,532)	-0.66 (-0.74, -0.56)	57.26 (34.57, 87.74)	9.35 (5.35, 14.92)	-5.93 (-6.12, -5.75)
DALYs	11,057 (8,057, 14,861)	8,138 (5,588, 11,594)	-0.26 (-0.50, 00.07)	126.72 (92.24, 171.14)	46.71 (32.29,67.1)	-3.51 (-3.83, -3.18)
**Maternal abortion and miscarriage**						
Prevalence	1,458 (896, 2,152)	2,731 (1,775, 3,919)	0.87 (0.52,1.32)	14.44 (8.94, 21.25)	14.1 (9.17, 20.19)	1.28 (0.32, 2.26)
Deaths	1350 (1815, 986)	46 (29, 69)	-0.97 (-0.98, -0.95)	15.27 (11.18, 20.73)	0.28 (0.18,0.41)	-13.63 (-14.02, -13.24)
YLDs	159 (76, 281)	301 (140, 508)	0.9 (0.12, 2.21)	1.57 (0.76, 2.74)	1.55 (0.74, 2.6)	1.32 (0.36, 2.30)
DALYs	78,156 (57,168, 105,005)	2,853 (1,914, 4,126)	-0.96 (-0.98, -0.94)	859.93 (628.7, 1162.03)	16.35 (10.9, 23.84)	-13.5 (-13.87, -13.12)
**Ectopic pregnancy**						
Prevalence	279 (174, 411)	188 (115, 284)	-0.32 (-0.42, -0.22)	2.9 (1.82, 4.23)	0.99 (0.6, 1.51)	-4.12 (-4.3, -3.94)
Deaths	7 (5, 9)	2 (1, 3)	-0.67 (-0.8, -0.48)	0.08 (0.06, 0.1)	0.02 (0.01, 0.02)	-6.32 (-6.88, -5.75)
YLDs	32 (16, 53)	22 (11, 37)	-0.32 (-0.42, -0.22)	0.34 (0.18, 0.56)	0.12 (0.06,0.2)	-4.12 (-4.30, -3.94)
DALYs	438 (324, 573)	157 (107, 214)	-0.64 (-0.77, -0.47)	4.51 (3.37, 5.95)	0.84 (0.58,1.15)	-6.09 (-6.60, -5.57)
**Indirect maternal deaths**						
Prevalence	NA	NA	NA	NA	NA	NA
Deaths	552 (395,727)	724 (507, 993)	0.31 (-0.16,0.98)	5.92 (4.27,7.85)	4.11 (2.85,5.58)	-0.66 (-1.17, -0.15)
YLDs	NA	NA	NA	NA	NA	NA
DALYs	33,098 (23,597, 43,599)	42,144 (29,445, 58,303)	0.27 (-0.19, 0.93)	347.19 (249.88, 457.63)	234.48 (164.00, 321.62)	-0.77 (-1.26, -0.15)
**Maternal deaths aggravated by HIV/AIDS**						
Prevalence	NA	NA	NA	NA	NA	NA
Deaths	0.01 (0.01, 0.01)	1 (0, 4)	745.41 (96.81, 11958.12)	0.01 (0.01, 0.01)	0.01 (0.01,0.03)	18.71 (12.8, 24.92)
YLDs	NA	NA	NA	NA	NA	NA
DALYs	0.05 (0.01, 0.3)	60 (3, 222)	692.94 (92.46, 10,701.35)	0.01 (0.01, 0.01)	0.36 (0.02, 1.31)	18.34 (12.37, 24.62)
**Late maternal deaths**						
Prevalence	NA	NA	NA	NA	NA	NA
Deaths	180 (121, 295)	139 (86, 209)	-0.23 (-0.56, 0.2)	2.03 (1.37, 3.26)	0.81 (0.5, 1.22)	-3.34 (-3.45, -3.23)
YLDs	NA	NA	NA	NA	NA	NA
DALYs	10,436 (6,921, 17,203)	7,844 (4,827, 11,719)	-0.25 (-0.57, 0.17)	115.09 (76.85, 187.47)	44.89 (27.66, 67.49)	-3.43 (-3.54, -3.32)
**Other maternal disorders**						
Prevalence	NA	NA	NA	NA	NA	NA
Deaths	166 (116.17, 228.88)	94 (60.58, 131.12)	-0.7 (-0.82, -0.53)	1.79 (1.26, 2.44)	0.53 (0.34, 0.74)	-3.84 (-4.08 to -3.6)
YLDs	784 (530, 1,082)	739 (486, 1,030)	-0.06 (-0.21, 0.12)	8.64 (5.84, 11.98)	4.04 (2.65, 5.63)	-2.62 (-2.78, -2.45)
DALYs	10,774 (7,695, 14,683)	6,210 (4,291, 8,449)	-0.42 (-0.63, -0.13)	113.43 (81.95, 152.88)	34.4 (23.8, 46.79)	-3.81 (-4.03, -3.58)

UI: Uncertainty interval; CI= Confidence interval; YLDs= Years lived with disability; DALYs= Disability-adjusted life years

* ASR = Age-Standardised Rate

** EAPC= Estimated annual percentage change; NA= Not availablez

**Table 2:** All age years lived with disability, disability-adjusted life years and ASR for neonatal disorders in Nepal, 1990-2019

Disease category and burden of disease metric	Number in 1990 (95% UI)	Number in 2019 (95% UI)	% Change from 1990-2019 (95% UI)	ASR^*^ 1990 (per 100,000) No. (95% UI)	ASR^*^ 2019 (per 100,000) No. (95% UI)	EAPC^**^ (95% CI)
**Neonatal disorders**						
Prevalence	303,146 (251,620, 358,917)	475,544 (414,572, 543,836)	0.57 (0.35, 0.86)	1194.65 (966.45, 1438.75)	1521.14 (1324.97, 1739.17)	0.98 (0.67, 1.29)
Deaths	25,212 (19,121, 31,381)	8,397 (6,752, 10,178)	-0.67 (-0.76, -0.54)	64.91 (49.27, 80.82)	28.49 (22.9, 34.53)	-2.61 (-2.87, -2.35)
YLDs	55,716 (38,885, 78,418)	117,301 (89,515, 148,158)	1.11 (0.65, 1.69)	229.87 (159.63, 325.7)	367.69 (280.48, 465.58)	2.3 (1.95, 2.65)
DALYs	2,295,550 (1,743,768, 2,846,364)	863,367 (714,585, 1,024,841)	-0.62 (-0.72, -0.48)	5,996.3 (4,566.29, 7,411.74)	2,898.61 (2,393.29, 3,434.7)	-2.29 (-2.49, -2.08)
**Hemolytic disease and other neonatal jaundice**						
Prevalence	7,635 (6,850, 8,502)	10,742 (9,587, 11,981)	0.41 (0.33, 0.49)	34.09 (30.54, 37.94)	34.02 (30.39, 37.92)	0 (-0.02, 0.03)
Deaths	3,584 (2,279, 5,250)	853 (545, 1,274)	-0.76 (-0.87, -0.57)	9.27 (5.91, 13.58)	2.89 (1.85, 4.32)	-3.48 (-3.85, -3.11)
YLDs	3,352 (2,517, 4,2689)	4,268 (3,210, 5,445)	0.27 (0.06, 0.52)	15.25 (11.45, 19.4)	13.53 (10.17, 17.26)	-0.41 (-0.43, -0.39)
DALYs	321,715 (205,135, 469,481)	80,043 (52,343, 117,293)	-0.75 (-0.86, -0.56)	838.32 (537.11, 1,220.36)	, 270.59 (176.61, 397.04)	-3.39 (-3.75, -3.03)
**Neonatal encephalopathy due to birth asphyxia and trauma**						
Prevalence	4,618 (2,429, 8,578)	33,257 (24,574, 43,788)	6.2 (3.15, 11.44)	18.83 (9.16, 36.67)	105.45 (78.09, 138.71)	6.38 (5.9, 6.85)
Deaths	6,392 (3633, 9421)	1,541 (873, 2,792)	-0.76 (-0.88, -0.56)	16.44 (9.33, 24.22)	5.23 (2.96, 9.47)	-3.74 (-4.2, -3.29)
YLDs	719 (212, 2535)	10,769 (6,790, 16,085)	13.98 (3.92, 43.52)	2.7 (0.68, 10.32)	33.92 (21.49, 50.62)	9.4 (9, 9.8)
DALYs	568,599 (323,241, 837,541)	147,687 (87,285, 258,868)	-0.74 (-0.87, -0.54)	1463.02 (832.78, 2,154.11)	498.35 (293.71, 875.51)	-3.53 (-3.95, -3.11)
**Neonatal preterm birth**						
Prevalence	304,224 (252,013, 363,964)	393,437 (337,023, 458,126)	0.29 (0.12, 0.52)	1205.43 (972.03, 1466.98)	1263.14 (1080.07, 1472.13)	0.21 (0.04, 0.38)
Deaths	3,873 (2,391, 5,966)	798 (463, 1,267)	-0.79 (-0.89, -0.6)	9.96 (6.16, 15.33)	2.71 (1.57,4.3)	-4.96 (-5.33, -4.59)
YLDs	45,856 (30,852, 66,522)	71,346 (52,932, 94,603)	0.56 (0.26, 0.99)	194.33 (130.30, 282.56)	, 221.64 (164.27, 293.97)	1.04 (0.81, 1.26)
DALYs	389,992 (258,029, 573,505)	142,278 (105,093, 189,150)	-0.64 (-0.77, -0.4)	1,079.5 (736.63, 1,541.85)	462.26 (337.65, 616.29)	-3.25 (-3.47, -3.04)
**Neonatal sepsis and other neonatal infections**						
Prevalence	13,490 (9,519, 18,431)	89,782 (67,812, 118,365)	5.66 (3.98, 8.05)	57.96 (40.2, 79.58)	286.53 (216.6, 377.08)	6.66 (6.12, 7.2)
Deaths	1,913 (910, 3,197)	1,192 (731, 1,892)	-0.38 (-0.71, 0.45)	4.93 (2.35, 8.23)	4.04 (2.48, 6.42)	-0.5 (-0.69, -0.31)
YLDs	871 (366, 1,955)	25,200 (16,090, 36,764)	27.92 (13.04, 62.21)	3.19 (1.32, 7.49)	79.63 (50.8, 116.21)	13.21 (12.6, 13.83)
DALYs	170,793 (81,885, 285,040)	131,105 (88,138, 189,152)	-0.23 (-0.61, 0.74)	441.14 (211.62, 734.9)	438.92 (294.18, 636.48)	0.21 (0.09, 0.34)
**Other neonatal disorders**						
Prevalence	NA	NA	NA	NA	NA	NA
Deaths	9,449 (6,364, 13,564	4,013 (2,877, 5,371)	-0.58 (-0.73, -0.31)	24.31 (16.39, 34.86)	13.61 (9.76, 18.22)	-1.6 (-1.89 to -1.3)
YLDs	4,918 (3,413, 6,647)	5,717 (4,282, 7,386)	0.16 (-0.09, 0.51)	14.40 (9.99, 19.53)	18.97 (14.21, 24.51)	1.38 (1.19, 1.58)
DALYs	844,451 (569,892, 1,210,754)	362,254 (260,842, 482,370)	-0.57 (-0.73, -0.31)	2,174.31 (1,470.4, 3,113.6)	1,228.50 (884.48, 1,636.03)	-1.57 (-1.86, -1.28)

UI= Uncertainty interval; CI= Confidence interval; YLDs= Years lived with disability; DALYs= Disability-adjusted life years;

* ASR = Age-Standardised Rate

** EAPC= Estimated annual percentage change; NA= Not available

## DISCUSSION

Despite improvements in the overall burden of maternal disorders and related deaths in Nepal over time, our study has identified an increasing number of maternal deaths and DALYs due to indirect causes and a sustained high burden of disease due to maternal haemorrhage. While the trend in prevalence decreased significantly for most subcategories, the increase in the prevalence of maternal abortion and associated YLDs highlights another key concern. From 1990 to 2019, the prevalence of neonatal disorders increased overall across all categories, with preterm birth (1263.14/100,000) and neonatal sepsis (286.53/100,000) being the most common in 2019. Neonatal sepsis and other infections had significantly the highest increase in prevalence trend. Death rates decreased for all subcategories, notably for preterm birth. The trend for YLDs increased for neonatal disorders overall except for haemolytic disease, and the most pronounced increase was observed for neonatal sepsis. The trend for DALYs decreased significantly except for neonatal sepsis. In 2019, other neonatal disorders and neonatal encephalopathy had the highest ASR DALY rates.

In Nepal, maternal health has improved due to the efforts of governmental and non-governmental organisations. Development of various policies, plans, and programs such as national health policy, safe motherhood policy 1998, long-term health plans, safe abortion policy 2002 including mobilisation of female community healthcare volunteers have addressed maternal and neonatal health issues (Supplementary Figure S5).^11^ Improvement in the uptake of recommended antenatal care (up from 9% in 1996 to 81% in 2022) and delivery care services at institutions (up from 8% in 1996 to 79% in 2022) have likely contributed to the reduction in burden metrics for major maternal disorders.^5^ However, maternal haemorrhage is still highly prevalent and had persistent impacts on deaths and DALYs in 2019. Targeted approaches, such as effective implementation and scale-up of the female community health volunteers delivered uterotonic medicine (misoprostol) during childbirth for haemorrhage control are needed.^12,13^

The unchanged prevalence and YLDs of maternal sepsis over time is concerning and warrants preventive measures to address modifiable risk factors for sepsis which include suboptimal maternal health, nutrition and hygienic practices. There are also infection risks related to the labour itself, including protracted labour, prolonged rupture of the membranes, multiple vaginal examinations, aseptic procedures and instrumental deliveries.^14^ In addition, although a large reduction in deaths due to maternal abortion (97% from 1990 to 2019) likely reflects the increased availability and effective utilization of abortion services, including task shifting to mid-level health professionals,^15^ the increased in prevalence (+87%) of unwanted pregnancies and associated YLDs (+90%) identifies an unmet need for effective contraceptives.

Our data confirm a transitioning towards indirect causes of maternal mortality as this had the highest ASR of deaths.^3,16,17^ Future maternal health programs should therefore focus on reducing these indirect deaths through improving care from pre-conception health and prenatal health screening stages. An important step will be to establish a national reporting mechanism to accurately identify and record indirect causes of maternal deaths to identify specific areas for action. Focusing on increasing access to skilled healthcare workforces with competence to handle normal and complicated childbirth and onward referral for care and comprehensive healthcare centres for marginalised populations and regions will also be important.^17-19^

Nepal's 2021 National Census revealed varying maternal mortality rates across provinces with Lumbini having the highest mortality of 207 per 100,000 live births, while Bagmati has the lowest, below the national average.^3^ Bagmati is the most advanced region in Nepal. It includes Kathmandu, Nepal's capital city, and it has more healthcare centres, a greater health workforce and better transport infrastructure, all of which may explain its lower maternal mortality rates. Our data indicate that greater investment in healthcare services is needed in provinces such as Lumbini. Local governments should invest in understanding the factors that contribute to the high maternal mortality rates in their regions so as to identify what targeted localised action is needed.

Abortion, a key reproductive right for women, if provided by unskilled personnel in unsafe, unsterile environments is also associated with infections, haemorrhages and deaths.^20,21^ The widespread provision of effective contraception should go together with safe abortion services to decrease burden of abortion and be a key future focus of the Nepali healthcare system.^22^ Nepal is at the third level of obstetric transition,^17^ where maternal mortality level falls between 299 to 50 per 100,000 livebirths, the fertility rate is comparatively lower to previous years, and a higher proportion of women are accessing healthcare. This presents a critical time to improve quality of care by ensuring provision of advanced delivery care, such as caesarean sections and instrument delivery, as indicated, and upskilling of existing birth attendants for tapping maximum benefit.^23,24^

The marked decline in deaths and DALYs of neonatal disorders may be attributed to the provision of skilled care during labour and delivery, antenatal corticosteroids for the management of mothers at risk of preterm birth, basic newborn care, managing and resuscitating non-breathing babies at birth, Kangaroo mother care for stable preterm and low birth weight babies, treatment of neonatal sepsis and inpatient supportive care for sick and small babies.^25^ Similarly, the decreased trend in deaths and prevalence rates particularly for haemolytic diseases may point to its effective management during childbirth.

However, we observed an increased trend of prevalence and YLDs of other neonatal disorders, possibly due to improved survival of neonates^26^ and better reporting due to an increasing proportion of institution-based births in Nepal. Similarly, the early identification and better management of medical complications with early induction and caesarean section may have contributed to decreasing stillbirth and increasing preterm births.^27,28^ Corroborating our observation, other countries have also reported an increasing prevalence of neonatal disorders, especially preterm births, that demands greater global investment in the health of women and newborns, particularly for low- and middle-income countries.^29,30^

This study reports the most comprehensive estimates of the burden of maternal and neonatal disorders and their subcategories in Nepal. We have described age-standardised changes over three decades, highlighting trends in disease burden and opportunities for Nepali health system improvement. We also recognise the study's limitations. While we have acknowledged geographical variations observed in maternal and neonatal mortality and their implications, we could not report disease burden at the sub-national level based on available data. Sub-national estimates are relevant for informing local policy decisions, which may differ by geographical region, including rural and urban areas, and the seven provinces with state-level governments. While the GBD Study 2019 has pooled data from available reports and published literature, it is possible that other local data sources were missed. The limitations of the GBD 2019 methodology have been described in detail elsewhere.^8^

## CONCLUSION

These data highlight priorities for future Nepalese national health program planning and enhanced investment in maternal and neonatal health given their unacceptably high burden. Specifically, given their observed prevalence and sustained impacts over three decades, maternal haemorrhage, indirect maternal deaths, maternal abortion and neonatal disorders are all areas that require urgent attention.
